# Internal validity evidence of a knowledge questionnaire on alcohol use, abuse and dependence and associated questions

**DOI:** 10.1590/0034-7167-2021-0377

**Published:** 2023-02-06

**Authors:** Divane de Vargas, Dionasson Altivo Marques, Rosa Jacinto Volpato, Erika Gisseth Leon Ramirez, Maria do Perpétuo Socorro de Sousa Nobrega

**Affiliations:** I Universidade de São Paulo. São Paulo, São Paulo, Brazil.

**Keywords:** Knowledge, Health Personnel, Alcohol-Related Disorders, Alcohol Drinking, Validation Study, Conocimiento, Personal de Salud, Trastornos Relacionados con Alcohol, Consumo de Bebidas Alcohólicas, Estudio de Validación, Conhecimento, Profissionais de Saúde, Transtornos Relacionados ao Uso de Álcool, Consumo de Bebidas Alcoólicas, Estudo de Validação

## Abstract

**Objectives:**

to perform content validity and verify the psychometric properties of the adapted version of an alcohol knowledge questionnaire and associated questions.

**Methods:**

a methodological study, in which a committee of judges analyzed the questionnaire item representativeness, clarity and relevance. Item response theory was used to assess the instrument psychometric properties applied to a sample of 240 health professionals.

**Results:**

the questions were adjusted according to judges’ assessment, obtaining a satisfactory Content Validity Index (0.98). High discrimination ability and adequate difficulty levels were observed in 75% of multiple-choice questions and in 25% of statements.

**Conclusions:**

the instrument presented content validity with satisfactory indices. However, it is recommended that the questionnaire adapted in this study be used in different samples of health professionals from other parts of Brazil, in order to provide greater robustness to its reliability.

## RESUMO

**Objetivos:**

realizar a validação de conteúdo e verificar as qualidades psicométricas da versão adaptada do questionário de conhecimentos frente ao álcool e questões associadas.

**Métodos:**

estudo do tipo metodológico, em que um comitê de juízes analisou a representatividade, clareza e pertinência dos itens do questionário. Utilizou-se a teoria da resposta ao item para avaliar as qualidades psicométricas do instrumento aplicado em uma amostra de 240 profissionais de saúde.

**Resultados:**

as questões foram ajustadas conforme avaliação dos juízes, obtendo um Índice de Validade de Conteúdo satisfatório (0,98). Observou-se alta capacidade de discriminação e níveis de dificuldade adequados em 75% das questões de múltipla escolha e em 25% das afirmativas.

**Conclusões:**

o instrumento apresentou validade de conteúdo com índices satisfatórios. Entretanto, recomenda-se que o questionário adaptado neste estudo seja utilizado em amostras diversas de profissionais de saúde, de outros locais do país, com o intuito de oferecer maior robustez à sua confiabilidade.

## RESUMEN

**Objetivos:**

realizar la validación de contenido y verificar las cualidades psicométricas de la versión adaptada del cuestionario de conocimientos sobre alcohol y preguntas asociadas.

**Métodos:**

estudio metodológico, en el que un comité de jueces analizó la representatividad, claridad y pertinencia de los ítems del cuestionario. Se utilizó la teoría de respuesta al ítem para evaluar las cualidades psicométricas del instrumento aplicado a una muestra de 240 profesionales de la salud.

**Resultados:**

las preguntas fueron ajustadas de acuerdo a la evaluación de los jueces, obteniendo un Índice de Validez de Contenido satisfactorio (0,98). Se observó una alta capacidad de discriminación y niveles de dificultad adecuados en el 75% de las preguntas de opción múltiple y en el 25% de las afirmativas.

**Conclusiones:**

el instrumento presentó validez de contenido con índices satisfactorios. Sin embargo, se recomienda que el cuestionario adaptado en este estudio sea utilizado en diferentes muestras de profesionales de la salud de otras partes del país, con el fin de darle mayor robustez a su confiabilidad.

## INTRODUCTION

Health professionals’ lack of knowledge has been identified as one of the main impasses related to screening for diagnosis or referral of individuals who use alcohol to specialized services in Brazil. Commonly, health workers who work in these care spaces have weaknesses in terms of assistance to people who have deleterious effects caused by abusive consumption of alcoholic beverages, as well as the strategies that facilitate the identification of resulting disorders^(1)^. Initiatives to address this situation have been developed on time, however little has been invested in the formal assessment of professionals’ knowledge regarding the theme, including to verify the impact of scarce interventions in these individuals’ knowledge^(2)^.

Consistent with these notes, a literature review, which investigated health professionals’ knowledge in the management of people with alcohol-related disorders and the commitment to biopsychosocial aspects related to the harmful use of this substance^(3)^, found that in the care practice of workers, there are barriers and difficulties in caring for this population, mainly due to the lack of knowledge to address the problem, carry out prior identification, offer interventions to change drinking behavior and referral to the health institution in the context of alcohol and drugs. Moreover, the authors pointed out that the lack of specific knowledge prevents compliance and interest of these professionals to promote the intervention, considering the problematic use of alcohol in an appropriate manner.

Studies that assessed the knowledge and skills in caring for individuals with alcohol-related disorders corroborate these findings, pointing out that there are difficulties in identifying patterns of use and in approaching this population^(4-5)^. Furthermore, investigations carried out with health professionals^(6-7)^ indicate that there is a lack of knowledge about the implementation of interventions that minimize the main impacts on the health of people who make problematic use of alcoholic beverages. However^(5)^, it is emphasized that, with training and experience in the area, professionals have better skills in these aspects.

In general, most studies that investigated, mainly, attitudes towards alcohol abuse and dependence and associated questions^(8-11)^, training and skills of professionals in the care of individuals with problematic use of this substance^(8,10)^, brings as outcome health professionals’ knowledge. Such studies show the importance of training and measuring knowledge during the training process, with a view to implementing motivational strategies for behavior change due to alcohol abuse, which can improve professionals’ attitude^(12,13)^, the quality of care provided and the interprofessional work in the care of this population^(3,12-13)^.

The measurement of health professionals’ knowledge about alcohol-related disorders can be performed using qualitative methods, such as observation, focus groups or individual interviews. However, these approaches require specific skills, are time-consuming and cannot be implemented on a large scale. In contrast, quantitative methods, such as the use of research questionnaires or standardized instruments, seem to be more appropriate for large-scale assessments or repeated assessments.

In a survey that sought to identify and assess the psychometric properties of instruments developed to assess knowledge regarding alcohol-related questions and associated questions^(14)^, 21 available questionnaires were identified that had the ability to assess health professionals’ knowledge in relation to alcohol abuse, with good results in terms of validity and reliability. Of these instruments, 20 were in English and one in French, and the existence of an instrument in Portuguese was not reported. Considering the results of the previous study^(14)^, a new search was performed in the electronic databases PubMed, Virtual Health Library, Scopus, Web of Science, LILACS, CINAHL, Embase, PsycINFO, which confirmed the absence of reliable instruments to measure knowledge about alcohol-related questions published in Portuguese. In view of this gap, a search was carried out in the gray, open grey and MedNar literature databases, on Google Scholar as well as in repositories of dissertations and theses.

In this search, a thesis developed at the *Universidade Federal de São Paulo* ^(15)^ was found, which assessed the impact of a course in diagnosis and treatment of alcohol abuse and dependence on Primary Health Care professionals’ knowledge, proposing the “questionnaire of knowledge regarding alcohol, alcoholism and associated questions”^(15)^, which was submitted to face validity in the same study by expert judges^(15)^. It was considered that this instrument^(15)^ was the only one found published that has been developed and validated in Brazilian Portuguese and, therefore, more suitable for application in the country when compared to the questionnaires identified in a previous study^(14)^, and that this did not have its psychometric properties exploited. It is considered appropriate to produce evidence of the instrument’ internal structure, which can support its use among Brazilian nursing and health professionals.

Given the great magnitude of alcohol-related problems in population’s health and the scarcity of formal education of nurses in this area, the availability of a reliable instrument has the potential to contribute both to practice and to teaching and research in this area by nurses. Thus, the availability of a standardized and valid questionnaire to identify health professionals’ knowledge regarding alcohol and associated questions is desirable, and can be of great importance to assess the effectiveness of continuing education programs in the area of alcohol and other drugs, in addition to identifying professionals’ knowledge in training after teaching strategies.

## OBJECTIVES

To perform content validity and verify the psychometric properties of the adapted version of an alcohol knowledge questionnaire and associated questions in a sample of health professionals.

## METHODS

### Ethical aspects

This study was approved by the Human Research Ethics Committee of the *Universidade de São Paulo* School of Nursing (CEP-EEUSP). All participants signed the Informed Consent Form.

### Study design

This is a methodological study, carried out according to the guidelines pointed out in literature and conducted in two stages: content validity^(16)^ and preliminary assessment of the questionnaire’s psychometric properties^(17)^.

### Knowledge questionnaire on alcohol and alcohol-related disorders

The questionnaire of knowledge about alcohol-related disorders^(15)^ originally consists of three multiple-choice questions and 15 statements to indicate “true” or “false”, distributed in 5 categories, namely: 1) diagnosis of harmful use and dependence^(15)^; 2) amount of low-risk use^(15)^; 3) diagnosis of mental illnesses or complications commonly associated with alcohol abuse (acute intoxication, withdrawal syndrome, alcoholic hallucinosis, cognitive impairments, and Wernicke-Korsakoff syndrome^(15)^; 4) pharmacological treatment of dependence and complications^(15)^; and 5) brief intervention^(17)^. The original questionnaire was used to assess changes in knowledge after educational intervention among health professionals working in the Primary Health Care network (physicians, nurses, nursing technicians, social workers, psychologists and health workers)^(15)^.

The version used in this study was adapted with the prior authorization of the questionnaire’s author^(15)^, in order to adapt the questionnaire for use with health professionals in general without specific training in the subject, but who can provide assistance and care to individuals who abuse alcohol. When adapting the instrument, 7 statements were excluded from the “false” or “true” section, statements 1, 4, 11, 12, 13, 14 and 15, referring to the category pharmacological treatment of dependence and complications. These statements were excluded, since they essentially involved aspects related to drug prescription, which is the prerogative of medical professionals. In addition to these excluded items, the answer option for question 1, “He is using 35 IU of alcoholic beverages, enough amount to have a diagnosis of dependence”, was also excluded, since the diagnosis is also a medical prerogative.

In order to make it more comprehensive from the point of view of the scope of professions in health, the 3^rd^ answer option of question 3 was also eliminated, “It is a pathological intoxication and the treatment is intravenous glucose, but always with thiamine for avoid Wernicke-Korsakoff Syndrome”, as it is a treatment that requires a medical prescription. The other three answer options for this question were maintained, preserving only the excerpt that mentions the types of intoxication (pathological, acute and chronic). A multiple-choice question was added that presents four alternatives as an answer, only one of which is true (question 4), related to tolerance, “the process of adaptation of an organism to the use of a psychoactive substance”, characterized by the decrease in the response to the administration of a certain dose, which typifies that the individual needs to progressively increase the consumption of psychoactive substances in order to obtain the desired effect. It is considered one of the signs present in alcohol abuse^(18)^ and that should be considered by professionals when assessing pattern of use (“When a person says they need more and more alcohol or another drug to “be high”, this can demonstrate”)^(19)^. After the adaptation changes were completed, the questionnaire resulted in a configuration of four questions with only one correct answer and eight statements to mark “true” or “false”^(19)^, totaling 12 items, distributed in the following categories:

*Pattern of use (diagnosis of harmful use and dependence and amount of low-risk use)* ^(15)^: the pattern of alcohol use is defined by the WHO as the relationship between the number of alcoholic beverages ingested and the frequency with which individuals consume alcohol. Pattern of use of is classified as consumption without health risks, risky consumption, harmful use of alcohol and people with possible alcohol-related disorders^(18)^. *Detection of complications:* comprises the diagnosis of mental disorders or complications resulting from the abusive use of alcoholic beverages, such as alcohol intoxication, defined as the alteration of cognitive functions and behavior by abusive consumption of alcohol. Depending on clinical picture intensity, it can result in coma, leading the individual to death^(18)^. *Psychosocial approaches (brief intervention)*: among the psychosocial approaches evidenced in the literature, brief intervention (BI) stands out, defined as a strategy capable of promoting changes in drinking behavior, helping to develop skills to minimize deleterious effects on people’s health^(20-21)^.

*Stages of readiness for change:* Prochaska and Diclemente^(22)^ described four motivational stages that people need to go through to achieve the change in drinking behavior, such as: *pre-contemplation*, a phase in which individuals have not yet reflected or there is no interest in changing behavior; *contemplation,* phase in which the person thinks about the possibility of changing his behavior, but there is still a lack of determination to change unhealthy habits; *preparation*, a phase in which the subject admits that harmful habits are causing negative implications and wants change; *action*, a phase in which people implement what they planned, but have not yet reached stability on their risk behavior; and *maintenance*, a phase in which the individual assumes a new behavior, seeking to remain in this purpose of readiness for change. It is worth noting that the person, after going through the referred stages of change, is at risk of relapse^(22)^.

### Data collection

This process consisted of two phases, content validity process and preliminary psychometric property assessment of a knowledge questionnaire on alcohol-related questions.

#### Phase 1 - Content validity process

This is a careful analysis of assessment instruments, carried out by a committee of professionals with experience in the area of additions to alcohol and other drugs^(16-17)^. The content validity of this questionnaire on knowledge about alcohol-related disorders was carried out by consulting the *Curriculum Lattes* Platform, available on the Brazilian National Council for Scientific and Technological Development (CNPq - *Conselho Nacional de Desenvolvimento Científico e Tecnológico*) portal. To complete the phase, a committee of judges, composed of six health professionals from different regions of Brazil, with academic training at doctoral level, chosen for their expertise in the area of mental health, with emphasis on alcohol-related disorders. The judges assessed the questionnaire through an instrument developed in software REDCap, between January and March 2021. The message sent contained instructions for filling in and scoring of representativeness, clarity of questions and statements, in addition to pointing out the relevance of each item in the four categories of the questionnaire: pattern of use, detection of complications, psychosocial approaches and stages of readiness for change. At the end of the assessment instrument, a space was also made available for the judges to make observations they deemed necessary about the items.

#### Phase 2 – Preliminary psychometric property assessment

In order to carry out this analysis, secondary data from a longitudinal study carried out between 2010 and 2016, involving a sample of 1,400 health professionals^(23)^, were used. Of these, n=240 (17%) responded to the knowledge questionnaire (Chart 1), composing the sample of this study. To guarantee a heterogeneous sample, participants worked at different levels of health care, such as public hospitals, Psychosocial Care Centers and Basic Health Units of Western São Paulo.

**Chart 1:** Items that have undergone modifications, as suggested by the committee of judges, São Paulo, São Paulo, Brazil, 2021

Item	Before judges’ assessment	After judges’ assessment
1	A 50-year-old patient has been using alcohol since the age of 20 Use distilled beverage (whiskey) every day: one dose (50 ml) at lunch and another (50 ml) at dinner. He is married and his wife often gets angry at the amount he drinks. In the last 5 months, he has been showing depressive symptoms, such as discouragement, loss of appetite and insomnia (__) Patient is dependent because he has been using alcohol for a long time and has marital problems due to this. (__) Patient meets criteria for diagnosis of harmful use. (__) Diagnosis is addiction because patient already has alcohol-related disorders. (__) I do not know.	A 50-year-old patient has been using alcohol since the age of 20 He drinks beer every day: two doses (700 ml) at lunch and another (350 ml) at dinner. He is married and his wife often gets angry at the amount he drinks. In the last 5 months, he has been showing depressive symptoms, such as discouragement, loss of appetite and insomnia (__) Patient is dependent because he has been using alcohol for a long time and has marital problems due to this. (__) Patient meets criteria for diagnosis of harmful use. (__) Patient uses low risk because he already has alcohol-related disorders. (__) I do not know.
5	A grown man can drink up to a maximum of 2 shots of whiskey (total of 100 ml) per day.	A grown man can drink up to a maximum of 2 cans of beer (total of 700 ml) per day.
9	The goal of (socially) safe drinking should be avoided for patients who abuse alcohol.	The goal of low-risk drinking should be avoided for patients who abuse alcohol.
10	For patients who are not motivated to treat, physicians must respect their opinion and wait until they feel motivated to start treatment.	For patients who are not motivated to treat, health professionals must respect their opinion and wait until they feel motivated to start treatment.
11	Patients in pre-contemplation consider quitting drinking, but cannot change their behavior in relation to drinking.	Patients in pre-contemplation consider quitting drinking, but still cannot change their behavior in relation to drinking.
12	Once alcohol dependence has been identified, the first guidelines should be given and patients referred to an expert.	Once alcohol dependence has been identified, health professionals must provide the first guidelines and refer patients to an expert.

*Source: Silva, 2005; Soares 2010.*

The assessment of psychometric properties was performed using the item response theory (IRT). This theory has the purpose of assessing item by item and the behavior of individuals in relation to the items, seeking to identify the factors that can affect each individual item, determining the probability of success or choice by individuals. According to this theory, the items are qualified according to the following parameters: i) item discrimination; ii) degree of difficulty of item^(24-25)^.

The interpretation of the IRT parameters is obtained by observing the Item Characteristic Curve (CCI)^(25)^. In discrimination analysis, the more the curve resembles an “S” shape, the more discriminative the question. Values can vary between 0 (very low discrimination) and 3 (high discrimination). To determine an item difficulty level, the point of intersection between the x axis (individual’s ability) and the y axis (probability) must be observed, so that the occurrence of correct answers is 50%, and the values obtained must behave between -3 (easy items) and +3 (hard items).

### Data analysis

Phase 1 data, originated during the content validity process, were stored in the REDCap software and, later, analyzed in the R software (version 3.6), to verify the agreement between the judges, using the Validity Index of Content (CVI). The data corresponding to psychometric property preliminary assessment were grouped in a database (SPSS), being analyzed in the R program (version 3.6), using the two-parameter logistic model, difficulty of questions and statements to mark “true” or “false” and ability to discriminate.

To calculate the CVI, a four-point Likert scale was used to assess clarity and representativeness, using the following formula: CVI = Number of answers “3” or “4”

### Total number of answers

Item relevance was assessed qualitatively. Each judge agreed about each question proposed in the questionnaire, indicating a dimension that they considered appropriate for the classification of analyzed questions.

### Sample characteristics

Of the 240 health professionals who completed the questionnaire, 47% (112) were nursing professionals (nurse, technician and nursing assistant), 21% (50) were psychologists and 32% (78) were other health professionals (physicians, occupational therapist, social worker and others). The average age of respondents was 37.6 years, with a minimum of 20 and a maximum of 65 years. Thus, 86% (207) were female and, of these, half declared themselves married (50%; 119).

## RESULTS

### Content validity

As a result of content validity, the six judges made some suggestions for changes, according to the representativeness, clarity and relevance of the questions and statements (Chart 1), which were analyzed in detail by the authors.

Among the observations pointed out by the judges, 50% of them indicated that question 1, “A 50-year-old patient has been using alcohol since the age of 20. He drinks hard liquor (whiskey) every day: one dose (50 ml) at lunch and another (50 ml) at dinner. He is married and his wife often gets angry at the amount he drinks. In the last 5 months, he has been showing depressive symptoms, such as discouragement, loss of appetite and insomnia”, would need a minor review regarding the response options: “Patient is dependent because he uses alcohol for a long time and has marital problems because of this”, “Patient meets criteria for the diagnosis of harmful use”, “Diagnosis is dependence, because patient already has alcohol-related disorders”, due to the similarity between answer options 1 and 3. All suggestions made by judges and changes made to the questionnaire items are described in Chart 1.

Although judges did not suggest changes in relation to question 2, “Patient with a diagnosis of alcohol dependence decreased his intake for 3 days and presents auditory hallucinations (voices of men calling his name). Does not present changes in consciousness level”, and their response options, “1) The diagnosis is AWS (alcohol withdrawal syndrome) and treatment is hospitalization with benzodiazepines and hydration”, “2) The diagnosis is Alcoholic Hallucinosis” and “3) The diagnosis is Delirium Tremens”, half of them (50%) considered, in the field available for observations, that the question and its response options had a high degree of difficulty for health professionals without specific training in the area of mental health. The judges (33.3%) also suggested changes in all questions that included “whiskey” as a type of drink, suggesting replacing it with beer, as they considered this to be the most consumed beverage in the Brazilian context (Chart 1). In addition to describing the definition of a standard dose, they specify this type of beverage, justifying that a health professional without specialization in this area may not be aware of this concept. The other items that make up the questionnaire were assessed by 100% of judges as clear, representative and relevant, with no suggestions for adjustments.

In order to establish a quantifiable and reliable measure of judges’ assessment in relation to the instrument items’ representativeness, clarity and relevance, the Content Validity Index (CVI) was used, which presented a total CVI for representativeness of 0.98 and a total CVI for clarity of 0.98. Table 1 shows the detailed values for each item.

**Table 1:** Results of a statistical test applied to content validity of a questionnaire on knowledge about alcohol-related disorders, São Paulo, São Paulo, Brazil, 2017

Item	Clarity		Representativeness	
	Mean (%)	CVI (%)	Mean (%)	CVI (%)
1	94.4	100.0	94.4	100.0
2	83.3	83.3	88.9	83.3
3	100.0	100.0	100.0	100.0
4	94.4	100.0	94.4	100.0
5	100.0	100.0	100.0	100.0
6	100.0	100.0	100.0	100.0
7	100.0	100.0	100.0	100.0
8	100.0	100.0	100.0	100.0
9	100.0	100.0	100.0	100.0
10	94.4	100.0	88.9	100.0
11	100.0	100.0	100.0	100.0
12	83.3	100.0	88.9	100.0

*CVI – Content Validity Index.*

Regarding relevance assessment of each item for each of the four categories that make up the questionnaire, it was observed that, of the 12 items, only statement 9, to mark “true” or “false”, there is divergence between judges in relation to the classification, presenting indication for three categories, being 33.3% standard of use, 16.7% as a stage of readiness, and 50% in psychosocial approaches. The other items were mostly classified in the following categories: consumption pattern (items 1 and 4 and true or false statements 5 and 6), readiness stage (statements 10 and 11); complications (items 2 and 3); and psychosocial approaches (statements 7, 8 and 12).

### Preliminary psychometric property assessment

Regarding the psychometric property preliminary assessment of a questionnaire on health professionals’ knowledge in relation to alcohol-related disorders, the results are presented according to the characteristics analyzed by the IRT, i.e., distribution of the number of correct answers among the 12 items. Noting that no participant got more than nine correct answers, 66% of participants corrected up to six questions, with items 1, 2, 5 and 10 being the ones that presented greater difficulty, showing higher percentages of error.

### Ability to discriminate items

The analysis of ability to discriminate multiple-choice items proved to be high for items 1 (1.45), 3 (0.92) and 4 (0.79), and moderate for item 2 (0.19). In statements 5 (0.87) and 8 (0.89), moderate discrimination was observed, and in items 7 (0.12), 9 (0.25) and 10 (0.08), the analysis showed low discrimination capacity. Regarding statements 6, 11 and 12, they presented negative discrimination coefficients.

### Difficulty of item levels

**Figure 1:**
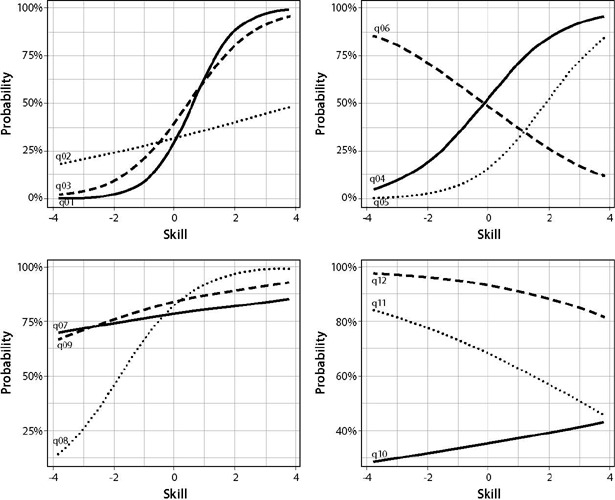
Characteristic curves for the difficulty level of multiple-choice items 1 to 4 and characteristic curves for the difficulty level of statements “true” or “false” 5 to 12, São Paulo, São Paulo, Brazil, 2017

Regarding difficulty level, multiple-choice items 1 (0.62), 3 (0.46) and 4 (-0.20) indicated adequate levels of difficulty; however, item 2 (4.17) was outside the expected range of [-3; +3], indicating that the probability of hitting varies little, depending on the individual’s skill. This situation was also observed in the statements to mark “true” or “false” 7 (-10.99), 9 (-6.60), 10 (7.16), 11 (3.13) and 12 (8.71), with statements 5 and 6 being the only ones in the category that presented adequate levels of difficulty, (1.86) and (-0.14), respectively. Figure 1 presents the characteristic curves related to the difficulty level of each multiple-choice item (1 to 4) and of each statement (5 to 12).

## DISCUSSION

This study aimed to carry out content validity and verify the psychometric properties of the adapted version of a questionnaire of knowledge about alcohol and associated questions, proposed by Silva^(15)^, for application among health professionals. As recommended by experts in the field, six judges were consulted. There was a variation of agreement in the assessments carried out, between 80% and 100%, regarding item representativeness, clarity and relevance, which is considered sufficient for maintenance of questions and statements to mark “true” or “false” for future analyses, since values lower than 70% would be considered for alteration or exclusion^(16)^.

**Chart 2:** Final version of an alcohol knowledge questionnaire and associated questions, obtained from the content validity and preliminary assessment of its psychometric properties in health professionals, São Paulo, São Paulo, Brazil, 2017

Item	Final version with adjustments
1	A 50-year-old patient has been using alcohol since the age of 20 He drinks beer every day: two doses (700 ml) at lunch and another (350 ml) at dinner. He is married and his wife often gets angry at the amount he drinks. In the last 5 months, he has been showing depressive symptoms, such as discouragement, loss of appetite and insomnia (__) Patient is dependent because he has been using alcohol for a long time and has marital problems due to this. (__) Patient meets criteria for diagnosis of harmful use. (__) Patient uses low risk because he already has alcohol-related disorders. (__) I do not know.
2	Patient diagnosed with alcohol dependence, reduced intake for 3 days and has auditory hallucinations (voices of men calling his name). There are no changes in consciousness level. (__) The diagnosis is AWS (alcohol withdrawal syndrome) and the treatment is hospitalization with benzodiazepines and hydration. (__) The diagnosis is Alcoholic Hallucinosis. (__) The diagnosis is Delirium Tremens. (__) I do not know.
3	Patient diagnosed with alcohol dependence was admitted to the emergency room with intense agitation after drinking a large amount of alcohol. (__) This is pathological intoxication. (__) This is acute intoxication (__) This is chronic intoxication. (__) I do not know.
4	When a person says they need more and more alcohol or another drug to “be high” this may demonstrate: (__) Tolerance. (__) Intoxication. (__) Dependence. (__) Harmful use.
5	( ) A grown man can drink up to a maximum of 2 cans of beer (total of 700 ml) per day.
6	( ) Women can drink less than the man, because she has proportionally more body fat, which increases alcohol bioavailability.
7	( ) Brief Intervention (BI) is a type of non-pharmacological treatment that is effective for mild dependencies and harmful users.
8	( ) BI can be applied in 10 to 15 minute consultations by physicians, nurses, psychologists, and other trained professionals.
9	( ) The goal of low-risk drinking should be avoided for patients who make harmful use of alcoholic beverages
10	( ) For patients who are not motivated to treat, health professionals must respect their opinion and wait until they feel motivated to start treatment.
11	( ) Patients in pre-contemplation consider quitting drinking, but still cannot change their behavior in relation to drinking.
12	( ) Once alcohol dependence has been identified, health professionals must provide the first guidelines and refer patients to an expert.

*Source: Silva, 2005; Soares 2010.*

Question 1 and statement 5 were considered to have an adequate difficulty and discrimination levels to assess the pattern of use. However, according to judges’ assessment, the questions and statements were readjusted and started to be presented, as illustrated in Figure 1.

Considering that this version was adapted with the aim of adapting the questionnaire to the use, abuse and dependence of alcohol in health professionals in general, the word “physician” was replaced by “health professionals” in all questions and statements that make specific reference to this professional category, which was also suggested by the committee of judges.

Judges’ analysis regarding statement relevance for the dimensions/categories previously defined showed that item 9, “The goal of safe drinking (socially) should be avoided for patients who make harmful use of alcoholic beverages”, was classified mostly by them in the categories of psychosocial approaches. However, considering the standard definition of low-risk use, according to WHO^(20)^, the authors agreed to keep this item in the standard category of use, even though it is directly related to psychosocial approaches. Moreover, “safe drinking” was replaced by “low-risk drinking”, according to available scientific evidence^(26)^.

Health professionals’ knowledge about patterns of use and techniques for early identification of problematic substance use in different areas of health care is important. International studies corroborate this perspective, pointing out that health promotion and prevention can improve the attitude of professionals^(12-13)^, the quality of care provided and interprofessional work in this area of care^(3,12- 13)^. Despite the low levels of difficulty and discrimination for item 9, “The goal of (socially) safe drinking should be avoided for patients who make harmful use of alcoholic beverages”, it was decided to keep it in the questionnaire, since its content meets the challenges in raising professionals’ awareness of the theme of harm reduction as a care, as has been pointed out by some authors^(27)^.

The assessment of knowledge about alcohol-related disorders is fundamental, as well as the identification of skills to provide care to users with alcohol-related disorders. To this end, the capacity of the items used in discrimination of such skills must be analyzed, identifying which questions/statements would be the most difficult and which the most discriminating, that is, which would require a higher level of knowledge and which would have a greater ability to discriminate whether a participant actually has mastery of the subject or not.

In this sense, question 2 was pointed out in the observations made by 50% of judges as a question with a high degree of difficulty for health professionals without specific training in the area, which was corroborated by the high percentages of error of respondents, for presenting an index void of skill discrimination. Therefore, it was suggested to carry out a more in-depth assessment before considering eliminating or replacing this question, as it is an item that addresses the importance of knowing psychopathological symptoms in the care of individuals who abuse alcohol^(28)^. Psychological and organic symptoms can generate confusion during diagnosis, especially among professionals who did not have adequate or sedimented access to knowledge correctly, in order to be able to differentiate and provide care properly^(29)^. On the other hand, the assumption that the item presents high difficulty for general practitioners may constitute an item with potential for studies aimed at comparing knowledge between samples from different extracts of health professionals, for example, general practitioners versus experts.

In statements 6, 7, 9 and 10, 11 and 12, the levels observed in the difficulty parameters and discrimination had not presented assessment capacity. This difficulty of discrimination enters the citizens can be related to the inherent factors to the proper structure of the question. To this respect, a study^(30)^ points that the presence of ambiguity in the writing of a question or even of answer alternatives, as well as grammar complexity, can increase difficulty level. Thus, in future tests, it can be presented the written item in different way so that if it can have evidences on this possibility. On the other hand, such items belong to the categories psychosocial approaches and pattern of use, themes that have already been evidenced in scientific production^(4)^ as deficient in the training of health professionals.

It is important to consider that the proposed questionnaire does not cover all the knowledge available in this area. However, it includes in its dimensions the general knowledge expected of nurses and other health professionals who work in non-specialized services. The dimensions included in this questionnaire are consistent with what the International Nurses Society on Addictions establishes, which recommends that every nurse must have minimum skills to track, identify and develop BI for harmful use of alcohol, regardless of their specialty or workplace^(31)^. Thus, the proposed instrument has the potential to access the training of these professionals to act efficiently in dealing with this phenomenon in line with contemporary approaches to understanding and coping with the problem.

### Study limitations

A gold-standard instrument to validate the results found in this study is not available in the literature. In addition, the data used for analysis come from observations carried out in a sample from a single region, which may have influenced the results, preventing generalizations. Although original, the data analyzed were extracted from a broader database resulting from a primary study completed in 2016. However, the object of the current study did not change its alignment with the theoretical and cultural model recommended by international guidelines in the last 10 years^(18,20,21,31)^. Still, aiming at ensuring the alignment of latent variables with the current references and minimize possible limitations, a new content validity carried out by researchers and professionals working in the area of the questionnaire was carried out between January and March 2021, allowing us to verify that, according to experts, the questionnaire content remained updated and relevant, therefore, with the potential to produce reliable evidence of the proposed instrument’s internal validity for use among nurses and other health professionals.

### Contributions to health

The main contribution is to provide a validated tool to assess knowledge in relation to the use, abuse and dependence of alcohol and questions related to health services, thus filling a gap in the national literature, since these assessments are usually not performed with validated instruments^(32)^ nor in Portuguese^(33)^.

The questionnaire can also be an instrument to measure part of health professionals’ knowledge, before and after training interventions and continuing education, featuring a useful tool to guide the development of contents addressed in them, from categories capable of measuring knowledge in a continuum, which goes from detection of problematic use of alcohol to possible approaches in any care space, regardless of the professional category.

The provision of a reliable questionnaire also has the potential to contribute to the formulation of robust research, with the use of an instrument, which has the ability to assess and identify the main gaps in professionals’ knowledge, thus enabling the elaboration of proposals to improve training. in different teaching instances. On the other hand, the use of a standardized questionnaire may act as a tool for selecting individuals with greater ability to care for this population in selection processes. Professionals’ level of knowledge is directly associated with more positive attitudes towards individuals with alcohol-related disorders, and that such attitudes are considered precursors of quality care for this population.

## CONCLUSIONS

The adapted version of a questionnaire on knowledge about dependence and alcohol-related disorders presented satisfactory preliminary evidence of validity for an instrument under development, which allows new perspectives from the improvement and use of this questionnaire in the assessment of continuing education programs in the area of alcohol and other drugs, in addition to identifying professionals’ knowledge in training in situations of pre and post-teaching strategies. It is suggested that further investigations be developed in order to improve this assessment tool, in order to provide a tool with robust and favorable assessment properties to be applied among health professionals at different levels of care. It is recommended that the questionnaire adapted in this study be used in several samples of health professionals from other locations in the country, in order to offer greater robustness to its reliability.
